# *MET* aberrations and c-MET inhibitors in patients with gastric and esophageal cancers in a phase I unit

**DOI:** 10.18632/oncotarget.1828

**Published:** 2014-03-16

**Authors:** Denis L. Fontes Jardim, Debora de Melo Gagliato, Gerald S. Falchook, Filip Janku, Ralph Zinner, Jennifer J. Wheler, Vivek Subbiah, Sarina A. Piha-Paul, Siqing Fu, Mariela Blum Murphy, Jaffer Ajani, Chad Tang, Kenneth Hess, Stanley R. Hamilton, Sinchita Roy-Chowdhuri, Razelle Kurzrock, Funda Meric-Bernstam, David S. Hong

**Affiliations:** ^1^ Department of Investigational Cancer Therapeutics (Phase I Clinical Trials Program), The University of Texas MD Anderson Cancer Center, Houston, USA; ^2^ Department of Gastrointestinal Medical Oncology, The University of Texas MD Anderson Cancer Center, Houston, USA; ^3^ Department of Radiation Oncology, The University of Texas MD Anderson Cancer Center, Houston, USA; ^3^ Department of Biostatistics, The University of Texas MD Anderson Cancer Center, Houston, USA; ^4^ Department of Pathology and Laboratory Medicine, The University of Texas MD Anderson Cancer Center, Houston, USA; ^5^ Department of Medicine, University of California, San Diego, USA

**Keywords:** MET mutation, MET amplification, esophageal cancer, gastric cancer, c-MET inhibitor

## Abstract

We sought to investigate the demographics and tumor-associated features in patients with gastroesophageal (GE) malignancies referred to our Phase I Program who had formalin-fixed, paraffin-embedded tissue from archival or new biopsies tested for *MET* mutation and/or amplification. *MET* amplification was found in 5 of 76 (6.6%) patients (3/34 [8.8%] esophageal, 2/26 [7.7%] gastric and none in 22 gastroesophageal junction cancers). The only *MET* mutation detected in 3 of 41 (7.3%) patients was N375S. No demographic and histologic characteristics were associated with specific *MET* abnormalities. Median overall survival was 3 and 5 months for patients with and without a *MET* alteration, respectively (hazard ratio [HR] = 2.1; 95% CI, 0.8 to 5.5; P=.14). Sixteen of 81 (20%) patients were enrolled in a c-*MET* inhibitor trial. Best responses were stable disease in 3 patients (19%), including a patient with esophageal adenocarcinoma that remained on the trial for 9.9 months (wild-type for *MET* abnormality). All tumors with *MET* abnormality (n=3) progressed on a c-*MET* inhibitor in fewer than 2 months. In conclusion, *MET* abnormalities can be found in a small group of patients with GE adenocarcinoma and further studies are necessary to better characterize the prognostic and predictive impact of *MET* alterations.

## INTRODUCTION

Cancers of the upper digestive system are a global burden.[1] The prognosis of individuals with advanced esophageal, gastroesophageal junction (GEJ) and gastric cancer is poor[2, 3], and the development of new treatment strategies is an unmeet need. The approval of trastuzumab based on an overall survival benefit in a pre-selected patient population harboring overexpression of HER-2, is a recent advance in the treatment of gastric and gastroesophageal (GE) cancer.[4] Genomic sequencing of these tumors suggests that exploring molecular aberrations in selected patients may offer new avenues for targeted therapeutic opportunities.[5, 6].

*MET*-positive GE cancer is a promising molecular subtype, particularly as a potential target for c-MET inhibitors. *MET* encodes a tyrosine kinase receptor whose activation is involved in cancer progression.[7, 8] c-MET is physiologically activated by its natural ligand, hepatocyte growth factor (HGF)[9]. Paracrine HGF-induced activation of c-MET plays in important role in the pathogenesis of gastric cancers.[10] Moreover, *MET* gene amplification is one of the well-recognized mechanisms of c-MET overexpression and constitutive activation of *MET*/HGF pathway [11], and has been reported in 2% to 10% of GE adenocarcinomas.[12, 13] The results of the same studies showed that *MET* positivity is an independent factor for poor survival regardless of disease stage. In agreement with this observation, *MET* amplified tumors display a higher pathologic grade and present at a more advanced stage.[12] Taken together, this data suggest that c-MET is an important target in GE cancers.

Although far less frequent, *MET* mutations have also been described as a mechanism for c-MET pathway activation in gastric cancer and in other malignancies.[14, 15] The recognition of this subset of GE cancer with its poor prognosis is important for referring affected patients to clinical trials with experimental therapies. Many c-MET inhibitors are currently in development and some of them showed activity for GE tumors.[16] In a recent case series, two out of four patients with *MET*-amplified GEJ cancers had some tumor shrinkage with crizotinib, a c-MET inhibitor.[12] Rilotumumab is a humanized monoclonal antibody against HGF, and, thus, can interfere with the interaction of HGF and c-MET preventing receptor activation. Early results with this drug in GE cancer showed prolonged survival for patients with high c-MET expression[17] leading to a phase III trial, which is currently accruing patients (NCT01697072). Similar results were obtained with onartuzumab, another monoclonal antibody blocking the c-MET pathway, and an ongoing phase III trial is enrolling a pre-selected patient population with high levels of c-MET expression (NCT01662869).

A way to better characterize *MET* genetic abnormalities in patients with advanced GE tumors is sorely needed, especially considering the wide availability of different c-MET inhibitors being assessed in clinical protocols. We sought to investigate the demographics and tumor-associated features in consecutive patients with GE malignancies referred to our Phase I Clinical Trials Program who had *MET* amplification/mutation testing. We also assessed the outcomes of patients with GE cancer who were included in protocols containing a c-MET inhibitor.

## RESULTS

### Patient characteristics

A total of 81 patients with advanced esophageal (n=36), GEJ (n=17) or gastric (n=28) cancers were evaluated for *MET* mutation/variant (41 patients) or amplification (76 patients). Thirty-six patients were tested simultaneously for both genetic abnormalities. Except for two patients with a neuroendocrine histology and one with squamous cell cancer, all remaining patients had adenocarcinoma. Median age at diagnosis was 56 years (range, 27-88 years). The median number of prior therapies was 2 (range, 0-5). Patient characteristics according to *MET* status are summarized in Table 1.

**Table 1:** Demographic, histologic and genetic characteristics of patients stratified by c-MET mutation and amplification status

Characteristic	Not mutated (n=38) (%)	Mutated (n=3) (%)	Not amplified (n=71) (%)	Amplified (n=5) (%)
Age At Diagnosis: Median (IQR)	51 (34-87)	48 (27-67)	56 (27-87)	60 (34-70)
Prior Therapies: Median (IQR)	2 (0-5)	2 (2)	2 (0-5)	2 (1-4)
Gender				
Male	27 (71)	1 (33)	58 (82)	4 (80)
Female	11 (29)	2 (67)	13 (18)	1 (20)
Ethnicity (%)				
Asian/Arabic	5 (13)	2 (67)	9 (13)	1 (20)
Black	0	0	0	0
Hispanic	5 (13)	0	10 (14)	0
White	27 (71)	1 (33)	51 (72)	4 (80)
Undefined	1 (3)	0	1 (1)	0
Diagnosis (%)				
Esophageal	13 (34)	1 (33)	31 (44)	3 (60)
GEJ	8 (21)	0	16 (22)	0
Gastric	17 (45)	2 (67)	24 (34)	2 (40)
Histology (%)				
Adenocarcinoma	38 (100)	3 (100)	68 (96)	5 (100)
Intestinal	3	1	2	2
Diffuse	10	1	16	1
Not classified	25	1	50	2
Squamous Cell	0	0	1 (1)	0
Neuroendocrine	0	0	2 (3)	0
Grade				
Well differentiated	0	0	0	0
Moderately differentiated	15 (39)	2 (67)	32 (45)	2 (40)
Poorly differentiated	22 (58)	1 (33)	37 (52)	3 (60)
Not evaluated	1 (3)	0	2 (3)	0
Metastasis (%)				
# Met Sites – median (range)	2 (1-5)	2 (1-3)	3 (1-6)	2 (2-5)
Liver	18 (47)	2 (67)	39 (55)	3 (60)
Lungs	12 (32)	0	12 (17)	2 (40)
Bone	8 (21)	0	13 (18)	1 (20)
CNS	3 (8)	0	4 (6)	0
Peritoneum	19 (50)	2 (67)	31 (44)	2 (40)
Lymph nodes	24 (63)	2 (67)	46 (65)	4 (80)
Tissue of analysis				
Primary	28 (74)	2 (67)	24 (34)	2 (40)
Metastasis	10 (26)	1 (33)	47 (66)	3 (60)
Concomitant aberrations (%)				
Her-2 positivity	5/37	0/3	8/60	1/4
PIK3CA	3/36	0/3	5/54	0/4
KRAS	0/32	0/3	2/44	0/3
EGFR	1/31	0/2	1/36	0/3
P53	8/20	1/1	8/16	1/3
BRAF	1/32	0/3	0/38	0/3
NRAS	0/30	0/1	0/26	0/3
PTEN loss	2/21	0/2	3/39	0/1

### *MET* genetic aberrations

Five out of 76 (6.6%) patients had a *MET* gene amplification in FISH analysis (3 esophageal and 2 gastric cancers, all adenocarcinomas). The copy number of the *MET* gene in relation to CEP7 ranged from 3.11 to 16.4. A *MET* mutation/variant was detected in 3 out of 41 patients (7.3%). Of these patients, two had gastric and one had esophageal cancer. All mutations/variants detected were N375S, which has been previously reported as a polymorphism[18] (Table 2). *MET* amplification and mutation were mutually exclusive in patients simultaneously tested for both aberrations (n=36). The prevalence of *MET* mutation/variant and amplification was similar regardless what site of disease was tested (*MET* mutation, 7% vs. 9%; *MET* amplification, 8% vs. 6%, for primary vs. metastatic tissue, respectively)

**Table 2:** Histology and mutation status of patients with MET mutation and amplification, and their response to c-MET inhibitors

Patient No.	Diagnosis	Mutation/Copy Number	Concomitant Abnormalities	Best Response	PFS (mos)
Met mutated					
1	Esophageal	N375S	TP53	-	-
2	Gastric	N375S	-	-	-
3	Gastric	N375S	-	PD	1
Met amplified					
4	Gastric	4.2	TP53	PD	1.5
5	Esophageal	16.14	Her-2 overexp	-	-
6	Gastric	12	-	-	
7	Esophageal	3.11	-	PD	1
8	Esophageal	9.23	-	-	

### Comparison of clinical and mutational characteristics

No meaningful differences were detected in the age of diagnosis or number and pattern of metastatic sites among patients with *MET* mutation/amplification and wild-type patients (Table 1). There was a higher proportion of female and Asian individuals among patients testing positive for a *MET* variant (2 out of 3, 67%) although the numbers are too small for definitive conclusions to be drawn. The proportion of poorly differentiated tumors was similar in *MET* positive patients compared to wild-type as well (1 out of 3 for *MET* mutated versus 22 out of 38 for nonmutated and 3 out of 5 for *MET* amplified versus 37 out of 71 for patients with non-amplified *MET*). Few concomitant mutations were observed in the *MET* positive population: one patient with a *MET* variant and one with *MET* amplification had a concomitant TP53 mutation, while *MET* and HER-2 amplification were simultaneously detected in one patient (Table 2).

### Analysis of survival of *MET* positive patients and outcomes on Phase I protocols

**Figure 1:**
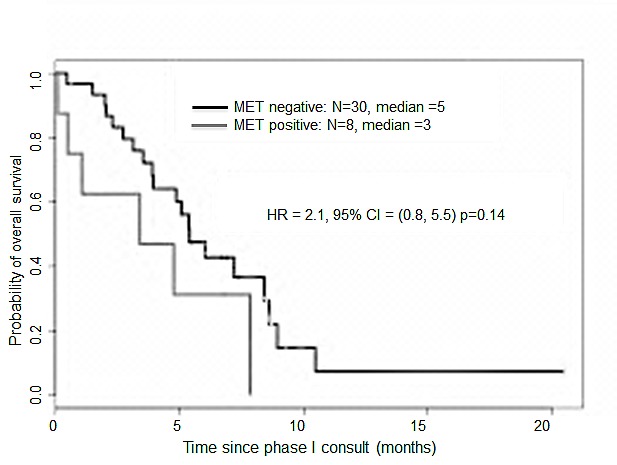
Kaplan-Meier overall survival curves for patients with gastroesophageal tumors according to *MET* status starting from presentation in a Phase I Clinic

Patients positive for either *MET* mutation/variant or *MET* amplification (*MET* positive group, n=8) were compared with patients wild-type for both abnormalities (*MET* negative group, n=30). Median OS from Phase I consult was 3 months versus 5 months for the *MET* positive and negative groups, respectively (HR for death = 2.1, 95% CI, 0.8 to 5.5, p=0.14; Figure 1).

**Figure 2:**
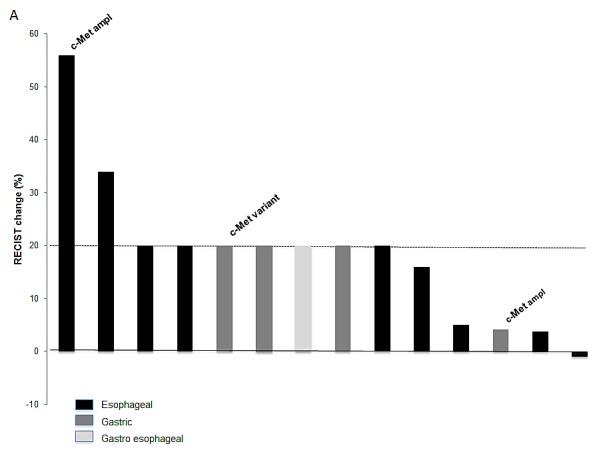

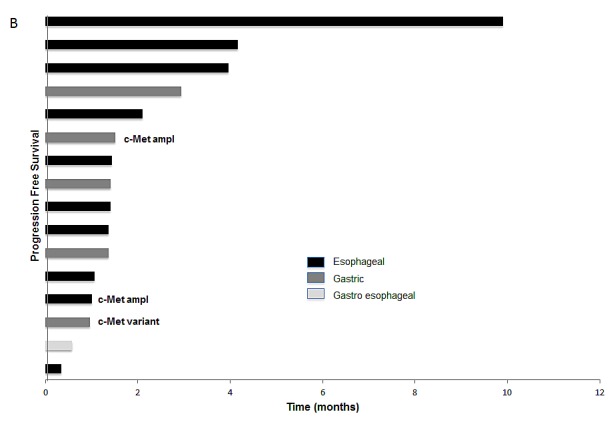
Waterfall plot showing responses (A) and PFS (B) of patients with gastroesophageal tumors treated on a phase I protocol including a c-MET inhibitor Patients harboring a *MET* genetic abnormality are indicated

Of the 81 patients included in this analysis, 44 (54%) were treated on at least one Phase I protocol. This proportion was 37.5% (3 out of 8) for *MET* positive patients and 56% (41 out of 73) for *MET* negative patients. Of note, the three patients with a *MET* abnormality were treated on a Phase I protocol containing a c-MET inhibitor. These patients had a PFS on these protocols of 1.0, 1.0 and 1.5 months (Table 2). Median PFS for the *MET* negative population on their first Phase I treatment regimen was 1.8 months (range, 0.26 to 9.9 months). Sixteen out of the 81 patients included in this study (20%) were enrolled on a protocol with a c-MET inhibitor. All of the c-MET inhibitors reported here were small-molecule inhibitors of the *MET* receptor tyrosine kinase (11 selective and 5 non-selective). No partial responses were detected and best responses were stable disease in three patients (Figure 2A). Median PFS was 1.4 months (range 0.33 to 9.9 months, Figure 2B) and all three patients with the greatest disease control rate had esophageal adenocarcinoma and were wild-type for a *MET* abnormality (Table 3).

**Table 3:** Histology and mutation status and response of patients treated with c-MET inhibitors

Patient No.	Histology	CMET abnormality	Other Mutations	Best Response	PFS (mos)
1	Esophageal	-	-	SD	4.0
2	Esophageal	-	-	SD	4.2
3	Esophageal	-	-	SD	9.9
4	Esophageal	-	Her2 ampl	PD	1.4
5	Esophageal	-	-	PD	1.4
6	Esophageal	-	-	PD	1.4
7	Esophageal	-	-	PD	1.1
8	Esophageal	-	-	PD	0.3
9	Esophageal	-	PTEN loss, TP53	PD	2.1
10	Esophageal	Amplification	-	PD	1.0
11	Gastric	Amplification	TP53	PD	1.5
12	Gastric	Variant (N375S)	-	PD	1.0
13	Gastric	-	PTEN loss, BRAF	PD	2.9
14	Gastric	-	-	PD	1.4
15	Gastric	-	-	PD	1.4
16	Gastroesophageal	-	Her 2 ampl TP53	PD	0.6

## DISCUSSION

Here we report *MET* amplification in 6.6% (5 out of 76) patients with advanced GE cancers referred to our Phase I department. *MET* mutation occurred at a similar frequency (7.3%), but was considered to be germline.[18] Our data are in line with previous series' reporting *MET* amplification in 2-7% of GE adenocarcinomas.[12, 19, 20] Although we included only one patient with a squamous histology, the prevalence of *MET* amplification in this histology is reported to be as low as 1%.[21]

The prevalence of *MET* amplification in GE cancer increased up to 5% with higher grade and more advanced disease in a previous report.[12] Considering that our population consisted mostly of patients with refractory and advanced disease, it is plausible to expect a higher prevalence of *MET* amplification compared to previous reports. However, we could not demonstrate any significant demographic and/or tumor associated features of *MET* positive tumors, including poor differentiation and an aggressive histology, as was previously reported for GE tumors and other malignancies.[12, 15] Selection bias is a possible explanation for this discrepancy, as patients referred to a Phase I unit generally have a reasonable performance status. Consequently, patients with a very aggressive phenotype may have been unwittingly excluded.

As expected, we detected a trend for a worse OS for *MET* positive GE cancers. Although this difference was not statistically significant, the small number of patients in this cohort may contribute to the lack of statistical power. We previously detected similar findings for patients with ovarian[22] and genitourinary cancers (submitted), suggesting that the aggressive phenotype conferred by *MET* genetic abnormalities is not dependent on tissue origin. In agreement with our results, patients with esophageal cancer and high c-MET expression had significantly reduced OS and disease-free survival in another series.[13] The OS of patients with advanced *MET*-amplified GE cancers was previously reported as 7.1 months[12]. Of note, in the cohort of patients in this study length of survival was assessed from the time of initial diagnosis. To avoid biased selection, we computed survival from the time of initial Phase I consultation and found an OS rate of 3 months. Considering that most patients had received several prior therapies (median of 2), this is an acceptable result.

It is somewhat challenging to enroll patients with relatively rare molecular abnormalities that are associated with a poor prognosis on clinical trials. Out of eight patients with *MET* abnormalities in our study, only three were included in trials with c-MET inhibitors. Other authors reported an even worse accrual. Notably, of 10 patients with *MET*-amplified GE cancers, none were included in a crizotinib trial.[12] Preclinical data suggest that *MET* amplified gastric tumors may be sensitive to c-MET inhibitors.[23] We report disappointing results for the three patients with *MET* abnormalities included in trials with these agents (1 patient with a *MET* variant and 2 with *MET* amplification). None of them had a response and their tumors invariably progressed in fewer than 2 months after starting treatment with a c-MET inhibitor. Similar findings were reported for two patients with *MET*-amplified gastric cancers treated with foretinib, a non-selective c-MET inhibitor.[24] Both were found to have progressive disease at the time of their first restaging assessment. Lennerz et al. reported that two out of four patients with *MET*-amplified GE tumors had rapid disease progression when treated with crizotinib. The other two patients had tumor reduction (up to 39%), although it did not last more than 4 months. Of note, responding patients had a *MET*/CEP7 ratio of more than 5 and both of our *MET*-amplified patients treated with a c-MET inhibitor had a ratio inferior to this. Interestingly, a recent series showed that the level of HER-2 amplification predicted sensitivity to trastuzumab.[25] In this study, a ratio of 4.7 was discriminative of sensitive patients, which suggests that a similar relation can be explored for *MET* amplification and sensitivity to c-MET inhibitors.

Additionally, one of the patients receiving a c-MET inhibitor in our series had a N375S *MET* variant. As demonstrated in preclinical models, this variant may confer resistance to c-MET inhibitors.[18] Clinical data suggested that this variant might decrease the risk of gastric cancer, probably through reduced affinity of HGF to the c-MET receptor.[26] Although similar prognostic implication was not confirmed in lung cancer, decreased cell death upon treatment with a c-MET inhibitor was showed in the presence of N375S variant.[27] In the light of our findings, the correlation of this variant and response to c-MET targeted agents is worth of further investigation.

Among all patients treated on trials with c-MET inhibitors in our series, the best result was stable disease for almost 10 months in a patient with esophageal adenocarcinoma who had received 3 prior therapies. Of note, this patient was negative for *MET* mutation and amplification, as well for other mutations tested (HER2, TP53, PIK3CA, PTEN, BRAF, c-KIT, ALK). Interestingly, encouraging preliminary results for treatment with onartuzumab and rilotumumab, which are antibodies against hepatocyte growth factor, were reported for GEJ and gastric cancers with a high expression of c-MET in immunohistochemical analysis[28], leading to two Phase III trials that are currently open for accrual. Taken together, these data suggest that further studies exploring different *MET* biomarkers simultaneously, such as via genetic and immunohistochemical analysis, is necessary to better understand predictive factors for response to c-MET inhibitors.

One of the weaknesses of this work is the lack of analysis of c-MET receptor expression levels, limiting some of our comparisons with previous studies correlating receptor overexpression with clinical and pathological features of GE cancers.[29, 30] The small numbers of patients harboring *MET* abnormalities in our series is another intrinsic limitation and precludes drawing definitive conclusions. Nonetheless, our results add to the current body of knowledge correlating the presence of *MET* genetic abnormalities with aggressive behavior and a worse prognosis in GE cancers. It is not clear if c-MET inhibition will offer reasonable antitumor activity for this population, but further exploration of biomarkers will likely clarify this issue.

## PATIENTS AND METHODS

### Patients

We retrospectively reviewed the electronic medical records of consecutive patients with advanced esophageal, GEJ and gastric carcinoma referred to the Department of Investigational Cancer Therapeutics at The University of Texas MD Anderson Cancer Center (MD Anderson) starting in May 2010 until March 2013. Patients were eligible for inclusion in data analysis if a primary diagnosis of any of these malignancies was confirmed and a tumor sample was sent to evaluate *MET* mutation or amplification status. This study and all associated treatments were conducted in accordance with the guidelines of the MD Anderson Institutional Review Board.

### Tissue samples and molecular analysis

*MET* mutation/variant and amplification were investigated in archival formalin-fixed, paraffin-embedded tissue blocks or material from fine needle aspiration biopsies obtained from diagnostic and/or therapeutic procedures. All histology was centrally reviewed at MD Anderson. *MET* mutation or variant analysis was performed in different Clinical Laboratory Improvement Amendment-certified laboratories as part of a gene panel analysis or in a single test. This included assessment of 182 genes using a targeted next-generation sequencing Foundation One platform (Foundation Medicine, Cambridge, MA), 46 genes in an Ion Torrent next-generation sequencing procedure (Baylor's Cancer Genetics Laboratory, Houston, TX) and 53 genes using a Sequenom Mass ARRAY platform (Knight Diagnostics, Portland, OR) or a PCR-based primer extension assay assessing mutational hot spots in the *MET* gene in the Division of Pathology and Laboratory Medicine at MD Anderson.

*MET* amplification was analyzed via fluorescence in situ hybridization (FISH) at MD Anderson or Baylor`s Cancer Genetic Laboratory. Copy numbers were expressed as gene copy number in relation to CEP7, a gene located near the centrosome of the same chromosome. *MET* was considered amplified when the *MET*/CEP7 signal ratio was ≥ 2.0 or when this ratio was < 2.0 but there were > 20 copies of *MET* signals and/or clusters in > 10% of the tumor nuclei counted.

### Treatment and evaluation

Patients referred to the Phase I Clinic who *MET* inclusion criteria were enrolled in clinical trials judged to be clinically appropriate by attending physicians. Treatment continued until disease progression, withdrawal of consent by the patient, clinical judgment deeming the necessity of removing a patient from a clinical trial, or development of unacceptable toxicity or death. Clinical assessments were performed as specified in each protocol, typically before the initiation of therapy and then at a minimum at the beginning of each new treatment cycle. Treatment response was assessed using computed tomography scans, magnetic resonance imaging and/or positron emission tomography scans at baseline before treatment initiation and then every 2 cycles (6-8 weeks). All radiographs were read in the Department of Radiology at MD Anderson and were reviewed in the Department of Investigational Cancer Therapeutics tumor measurement clinic. Responses were categorized using RECIST on the basis of specific protocol requirements[31, 32] and were reported as best response.

### Statistical analysis

Patient characteristics including demographics, tumor type, *MET* mutation and/or amplification status and associated genetic abnormalities were summarized using frequency distributions and percentages. Progression-free survival (PFS) was defined as the interval from the start of therapy to treatment discontinuation for disease progression or death related to disease progression. Overall survival (OS) was assessed using Kaplan-Meier curve analysis starting from the date of the first appointment in the Phase I Clinic.
